# Bacillin 20, a bacterial derived compound, improves soybean growth, photosynthesis and nutrients content under drought stress conditions

**DOI:** 10.1371/journal.pone.0332803

**Published:** 2025-10-21

**Authors:** Hamid Reza Eisvand, Donald L. Smith

**Affiliations:** 1 Department of Plant Production Engineering and Genetics, Faculty of Agriculture, Lorestan University, Khorramabad, Iran; 2 Department of Plant Science, McGill University, Montreal, Canada; Nuclear Science and Technology Research Institute, IRAN, ISLAMIC REPUBLIC OF

## Abstract

Water scarcity is a global challenge with profound implications, particularly for agriculture, where it undermines crop production by diminishing yields and heightening vulnerability to environmental stresses. This study investigates the impact of Bacillin 20, a derivative of *Bacillus thuringiensis*, on soybean plant physiology under drought stress, focusing on growth dynamics, photosynthetic activity, and nutrient assimilation. The experimentation was carried out using a factorial structure within a completely randomized design and four replications. Factors included drought levels (control, −0.75 MPa and −1.5 MPa) and Bacillin 20 concentrations (0, 10^−11^ M and 10^−9^ M). Results indicated that drought stress significantly reduced plant height, leaf area, shoot dry weight, photosynthetic rate, stomatal conductance, transpiration, substomatal CO_2_ concentration, nodulation, and root length and volume. Bacillin 20 application had mixed effects, with no significant impact on plant height but increasing leaf area, enhancing shoot dry weight under moderate drought, and improving photosynthetic rate. The interaction between drought and Bacillin 20 was significant, particularly in terms of shoot dry weight and photosynthetic rate. Additionally, Bacillin 20 at 10^−11^ M increased root tips by 12.6% and shoot dry weight by 28%; it increased nodule number by 51% only under normal moisture conditions, and decreased it under drought stress. Drought increased leaf N, Mg, Zn, Fe, Mn, and B contents, while Bacillin raised leaf N at −0.75 MPa and decreased Zn and Mn under severe drought (−1.5 MPa). The increased plant N and decreased nodulation under drought suggest enhanced nodule efficiency. Bacillin 20 did not affect P, K, Ca, and S contents, which were influenced solely by drought.

## Introduction

Oil and protein are essential components of human and livestock nutrition, with almost 70% of cooking oil and 50% of feed protein coming from plants. Among oilseeds, soybean (*Glycine max* L.) is particularly significant, contributing nearly 60% of global oilseed production and accounts for more than 25% of the protein consumption for food and animal feed worldwide, making it a leading commercial crop for vegetable oil and protein production [1].

With the increasing frequency of environmental challenges, particularly drought, ensuring stable crop productivity has become a major concern. Drought stress hampers plant growth, reduces photosynthetic activity [2], and disrupts nutrient uptake, leading to substantial yield losses [3]. As climate change intensifies, drought stress is expected to worsen, making it a more significant concern [4]. Consequently, identifying effective strategies to mitigate drought-induced crop losses is crucial. In this context, plant growth-promoting microbial derivatives have gained attention as a promising approach.

Agriculture, particularly fertilizer production, contributes significantly to greenhouse gas emissions. The application of plant growth-promoting microorganisms (PGPM) as biofertilizers can help crops resist environmental stresses like drought and salinity, while also reducing the reliance on chemical fertilizers and minimizing greenhouse gas emissions [5]. These microorganisms and their derived compounds as technologies enhance plant growth under both stressed and non-stressed conditions, promoting agricultural sustainability, especially in the face of climate change [6]. Recent studies have highlighted the potential of derived compounds from plant growth-promoting bacteria as effective biostimulants in sustainable agriculture. These compounds can enhance plant growth and stress tolerance, while avoiding the challenges associated with the survival of live inoculants in the field [7].

The beneficial effects of plant growth-promoting bacteria (PGPB) use can be categorized into direct and indirect mechanisms [8]. Direct mechanisms involve the production of substances that stimulate plant growth or biological fertility via the mobility of soil minerals [5]. Production of phytohormones, solubilization of phosphorus compounds, nitrogen fixation, production of siderophores, oxidation of sulfur compounds, production of ACC-Deaminase, are mechanisms of direct effects, and production of antibiotics, enzymes that destroy pathogen cell walls, improvement of plant systemic resistance, competition with pathogens, and production of volatile compounds, are the indirect mechanisms of PGPB effects on plants [9].

*Bacillus thuringiensis* NEB17 produces Bacillin 20 (formerly referred to as thuricin 17) [10]. Bacillin 20 is a microbe-to-plant signal compound isolated and characterized at McGill University [5]. Bacillus 20 has shown beneficial effects on plant growth under both optimal and stressful conditions, including soybean [6,11,12]. Bacillin 20 has been particularly effective under stressed conditions, a common scenario in field environments. Previous studies have demonstrated the positive impacts of PGPMs on plant growth, seed quality, and nutrient uptake, even under challenging environmental conditions like drought. [13,14].

The use of PGPB stimulating bacteria in bean improved the quality of seeds and seedlings, even in cases where the seeds were deteriorated, and ultimately increase grain yield. The best result was observed from simultaneous inoculation with *Rhizobium oligominusarum* and *Pseudomonas putida* [15]. Naamala, Msimbira [16] reported that a concentration of cell free supernatant (CFS) of 0.2% by volume from *Lactobacillus helveticus* EL2006H increased the germination of soybean by 44.37% in 100 mM NaCl salinity. However, despite the negative effect of salinity on root growth, the application of the inoculation liquid obtained from the above bacteria did not improve root growth. Subramanian, Ricci (6) reported that using the bacterial signal compounds lipo-chitooligosaccharide (LCO) and thuricin-17 (Th17), soybean seeds (variety Absolute RR) germinated more rapidly at salt stress levels of up to 150 mM NaCl. Plant growth promoting bacteria (*B. thuringiensis* NEB17) can help overcome deleterious effects of low root zone temperatures on nodulation and nitrogen fixation of *Glycine max* (L.) Merr. [11].

Application of *Pseudomonas putida* h-2–3 to soybean under drought and salt stresses stimulated the production of gibberellin and jasmonic acid, decreased abscisic acid and salicylic acid, and increased the activity of SOD and the amount of flavonoids produced due to stresses [17]. Soybean was less impacted by water stress when treated with *Bradyrhizobium japonicum* and thuricin-17 from *Bacillus thuringiensis*, so that thuricin-17 application under water stress increased plant biomass by 17%, accompanied with a 30% increase in root abscisic acid, and an increase of root length and of leaf water potential. In general, it improved nodule formation by 40%, caused a partial restoration of nodule-specific activity, nodule growth and consequently, an increase by 17% for total nitrogen in the plant. Overall, our findings reveal a new method to decrease the negative impact of water stress on crop plants. Results also demonstrate that the plant restored an adequate water and N balance by changing its root structure [18].

It has been found that biological fixation of nitrogen is not sufficient to achieve the highest possible protein content in soybean grain. Therefore, although soybean is a nitrogen-fixing plant, it requires supplemental N fertilizer to achieve maximum PSII efficiency, minimum chlorophyll fluorescence, and optimal yield [19].

This study explores the hypothesis that Bacillin 20 can enhance soybean growth and nutrient uptake under drought stress, aiming to provide a more sustainable and environmentally friendly approach to improving crop production.

## Materials and methods

### Experimental model and plant material

A factorial pot experiment was carried out structured to follow a randomized complete block design with four replications in a greenhouse of Plant Science Department of McGill University, from February 20 to March 28, 2024. Factors were drought stress levels (control, −0.75 MPa [megapascal] and −1.5 MPa) and Bacillin 20 concentrations (0, 10^−11^ and 10^−9^ M). The drought stress levels were set at −0.75 MPa for moderate stress and −1.5 MPa for severe stress, as established in previous studies [20,21]. Similarly, the selected Bacillin 20 concentrations were based on prior research demonstrating their efficacy in enhancing plant growth under stress conditions [22].

Untreated soybean (*Glycine max* L. Merr.) seed, variety B088Y1 was acquired from BREVANT Co. It is a Round Up Ready 2 Yield soybean variety with a 100-seed weight of 22.8 g.

Initially, seeds were surface-sterilized with 2% sodium hypochlorite for 5 min and washed with distillated water three times. Then, they were inoculated with *Bradyrhizobium japonicum* strain USDA 110 (CFU = 7.8 × 10^8^). After inoculation, three seeds were sown in each plastic pot (14 cm diameter and 15 cm height) containing Promix and watered with tap water. Seedlings were thinned to one a week after emergence.

Bacillin 20 was prepared in the Smith laboratory of McGill University. It was isolated from *Bacillus thuringensis* according Gray, Lee (10) and Subramanian, Souleimanov and Smith [23]. Bacillin treatments were applied as 10 mL root drenching one day after thinning (Growth stage = V1). Drought stress (osmotic water deficit stress) was applied two days after Bacillin treatment using polyethylene glycol (PEG) 8000 [24] as 270 mL of PEG solutions for the desired stress level for each treatment, with tap water at the control. During the experiment tap water was added equally to each pot. Half-strength Hogland solution (100 mL) was applied only one time at V3 stage to all pots. The greenhouse temperature was maintained at 23 ± 2°C, with a light intensity of 300 PPFD under a 16-hour photoperiod, and a relative humidity of 32%.

### Traits measurement

Photosynthetic rate, substomatal CO_2_, stomatal conductance and transpiration were measured with a LI-COR portable photosynthesis meter (LI-6400, USA) two weeks after treatment applications at V3. These measurements were conducted at 10:00–11:00 h on the middle trifoliate segment of a top-most fully developed trifoliate.

Plant height was measured before the end of experiment (R1 = beginning of flowering), then shoot parts were separated by cutting and measured for leaf area, shoot dry weight and leaf nutrient content. Leaf area was measured with a leaf area meter (LI-3100, LI-COR, Inc. USA). Subsequently shoot parts were oven dried at 70°C for two days and weighed. Leaves sample were sent to A&L Canada Laboratories Inc. for analysis of nutrient (N, P, K, Ca, S, Zn, Fe, Mg, Mn, and B) contents.

Roots were removed from the pots and washed carefully. Then they were scanned (Modified Epson Expression 10000XL, Regent Instruments Inc., Quebec, QC, Canada) at 400 dots per inch (dpi) resolution and the images analyzed by using WinRHIZO software (Reagent Instruments Inc.) to measure root length, root volume, root diameter, and root branching. Then nodule number was counted manually. Finally, the roots were oven dried (70°C for 2 days) for root dry weight measurement.

### Data analysis

All statistical analyses were performed using Minitab 16 software. The data were first examined for potential outliers using the Boxplot option (Simple). Normality was assessed using the Anderson-Darling test, and homogeneity of variances was tested using Levene’s test. When necessary, data transformation (e.g., square root transformation for shoot dry weight and log transformation for the number of nodules and transpiration rate) was applied to meet normality assumptions. The experiment was conducted in a factorial arrangement using a completely randomized design (CRD) with four replications.

A two-way analysis of variance (ANOVA) was performed using the General Linear Model (GLM) procedure to assess the effects of drought stress levels, Bacillin 20 concentrations, and their interaction on the measured traits. Mean comparisons were conducted using Tukey’s test at a 5% probability level. All analyses were performed under the fixed-effects model assumptions. Additionally, all graphs were generated in Minitab. Additionally, all graphs were generated in Minitab.

## Results

### Plant growth, morphology and dry weight

Drought stress significantly reduced plant height (*p* ≤ 0.01, Table 1), with both levels (−0.75 and −1.5 MPa) leading to a decline (Table 2). However, Bacillin 20 had no effect on plant height (Table 1). Similarly, leaf area decreased under drought stress (**p* *≤ 0.01, Table 1), with the highest leaf area observed in the control and the lowest at −1.5 MPa (Table 2). The application of Bacillin 20 (10^−11^ M) increased leaf area by 15.8% (*p* ≤ 0.05, Table 1, Table 3).

**Table 1:** Analysis of variance (mean square) of effects of drought stress and Bacillin 20 on some traits of soybean plants.

S.O.V	Df	Plant height	Leaf area	Shoot dry weight	Total root length	Root tips	Root diameter	Root volume	Root dry weight		Nodule number
Replication	3	251. 00 ^ns^	3679^*^	0.065^ns^	77732 ^ns^	323844^*^	0.010^ns^	0.600^ns^	0.006^ns^		0.171^ns^
Drought (D)	2	1069. 38^**^	104046^**^	3.755^**^	920530^**^	56804^ns^	0.069^**^	32.89^**^	0.359^**^		36.468^**^
Bacillin 20 (B)	2	16. 29 ^ns^	4518^*^	0.672^**^	4710^ns^	233229^*^	0.007^ns^	0.476^ns^	0.002^ns^		2.712^**^
D × B	4	41.20^ns^	928^ns^	0.199^**^	38840^ns^	74780^ns^	0.002^ns^	0.529^ns^	0.0006^ns^		1.478^**^
Error	24	93.85	1048	0.045	49864	46523	0.004	1.102	0.0074		0.098
Total	35										
S.O.V	df		Photosynthetic rate		Stomatal conductance		Transpiration			Substomatal CO_2_	
Replication	3		6.19^*^		0.0012^ns^		0.0013^*^			443^ns^	
Drought (D)	2		36.19^**^		0.0158^**^		0.0174^**^			10432^**^	
Bacillin 20 (B)	2		12.76^**^		0.00004^ns^		0.00005^ns^			2456^ns^	
D × B	4		0.66^ns^		0.0008^ns^		0.0007^ns^			3604^*^	
Error	24		1.750		0.0004		0.0003			1232	
Total	35										

*, **, and ns, indicate significant at 0.05, 0.01 probability levels, and non-significant, respectively.

**Table 2:** Effects of drought stress on some morphological traits of soybean.

Drought treatments	Plant height (cm)	Leaf area (cm^2^ plant^−1^)	Total root length (cm)	Root diameter (mm)	Root volume (cm^3^)	Root dry weight (g plant^-1^)	Leaf Mg content (%)
Control	51.92 ± 3.2^a*^	338.10 ± 11.4^a^	1593.33 ± 39.2^a^	0.69 ± 0.02^a^	5.67 ± 0.33^a^	0.71 ± 0.03^a^	0.31 ± 0.01^b^
−0.75 (MPa)	39.71 ± 3.3^b^	226.00 ± 10.6^b^	1202.57 ± 62.4^b^	0.58 ± 0.01^b^	3.43 ± 0.18^b^	0.368 ± 0.02^b^	0.46 ± 0.01^a^
−1.5 (MPa)	33.34 ± 1.8^b^	153.00 ± 11.3^c^	1057.94 ± 81.4^b^	0.54 ± 0.02^b^	2.51 ± 0.25^b^	0.250 ± 0.03^c^	0.45 ± 0.01^a^

*Grouping has been done using Tukey method at 5% probability level. Each value represents mean ± standard error. Means that do not share a letter are significantly different.

**Table 3:** Effects of Bacillin 20 on some morphologic traits of soybean.

Bacillin 20 treatments	Leaf area (cm^2^ plant^−1^)	Shoot dry weight (g plant^−1^)	Total root length (cm plant^−1^)	Root tips
Control	225.70 ± 22^b*^	1.692 ± 0.17^b^	1263.12 ± 71^a^	2219 ± 94^b^
10^-11^ M	261.30 ± 27^a^	2.160 ± 15^a^	1302.15 ± 89^a^	2498 ± 73^a^
10^−9^ M	230.20 ± 24^ab^	1.867 ± 13^b^	1288.60 ± 113^a^	2368 ± 67^ab^

*Grouping has been done using Tukey method at 5% probability level. Each value represents mean ± standard error. Means that do not share a letter are significantly different.

**Fig 1:**
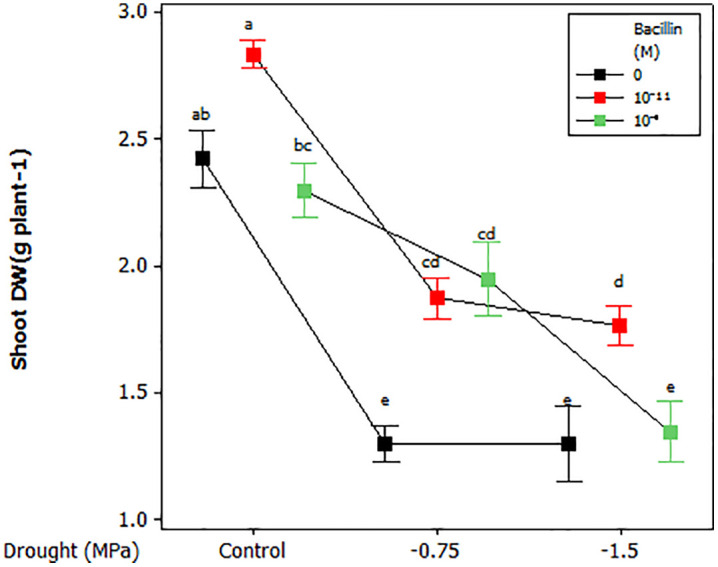
Effect of drought and Bacillin 20 on soybean shoot dry weight. Bars indicate standard error. Means that do not share a letter are significantly different, according to a Tukey test, at the 5% probability level.

A significant interaction between drought stress and Bacillin 20 was observed for shoot dry weight (*p* ≤ 0.01, Table 1; Fig 1). Under well-watered conditions, Bacillin 20 at 10^−11^ M increased shoot dry weight compared to 10^−9^ M, but neither exceeded the control. Under moderate drought (−0.75 MPa), Bacillin 20 improved shoot dry weight, while at severe drought (−1.5 MPa), only the lower concentration (10^−11^ M) had a positive effect (Fig 1). Drought stress also significantly reduced total root length (*p* ≤ 0.05, Table 1), with the shortest roots observed at −1.5 MPa, although differences between −0.75 MPa and −1.5 MPa were not significant (Table 2). Bacillin 20 had no effect on root length.

### Root traits

Root traits were variably affected by treatments. The number of root tips increased with Bacillin 20 (*p* ≤ 0.05, Table 1), with the highest count recorded at 10^−11^ M. A significant difference existed between the control and 10^−11^ M, while 10^−9^ M showed no difference from the control (Table 2). Drought stress significantly decreased root diameter (Table 1), with thinner roots observed under −1.5 MPa, though the difference between −0.75 MPa and −1.5 MPa was not significant (Table 2). Root volume followed a similar trend, decreasing significantly with increasing drought severity (*p* ≤ 0.01, Table 1, Table 2). Likewise, root dry weight was highest in the control and lowest at −1.5 MPa (Table 2).

### Nodule formation

**Fig 2:**
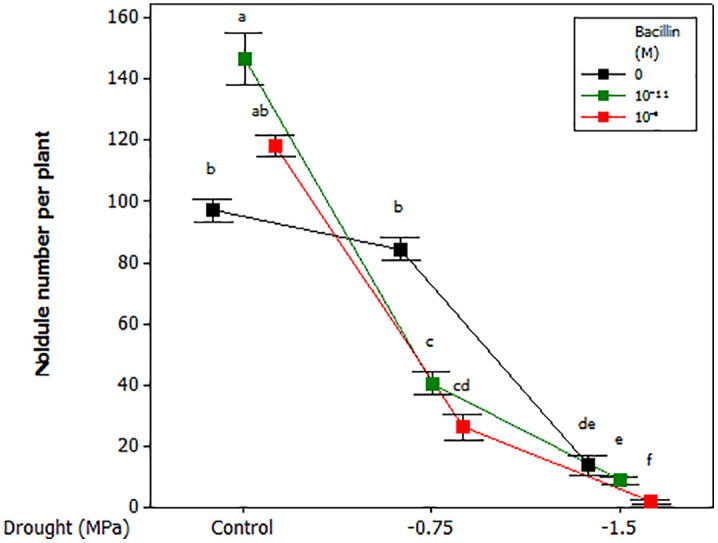
Interaction between drought stress and Bacillin 20 on soybean nodule number per plant. Means that do not share a letter are significantly different according to Tukey test with *p* ≤ 0.05. Bars represent standard errors.

**Fig 3:**
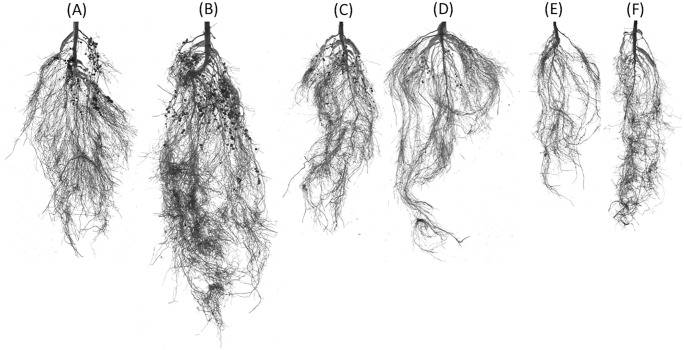
Root images taken from Bacillin 20 at 10^−11^ M that improves many features of root under a range of drought stress. **A:** Control + no Bacillin; **B:** Control + Bacillin 10^−11^ M; **C:** −0.75 MPa + no Bacillin; **D:** −0.75 MPa + bacillin 10^−11^ M; **E:** −1.5 MPa + no Bacillin; **F:** −1.5 MPa + Bacillin 10^−11^ M.

Nodule number was significantly influenced by the interaction between drought and Bacillin 20 (*p* ≤ 0.01, Table 1). Under well-watered conditions, Bacillin 20 (10^−11^ M) increased nodule number. However, at −0.75 MPa, Bacillin 20 application reduced nodule number, with both concentrations (10^−11^ and 10^−9^ M) showing similar effects. At −1.5 MPa, Bacillin 20 (10^−9^ M) reduced nodule number compared to the control and 10^−11^ M (Fig 2). Representative images of nodule frequency and root architecture are shown in Fig 3.

### Photosynthetic rate and gas exchange parameters

**Fig 4:**
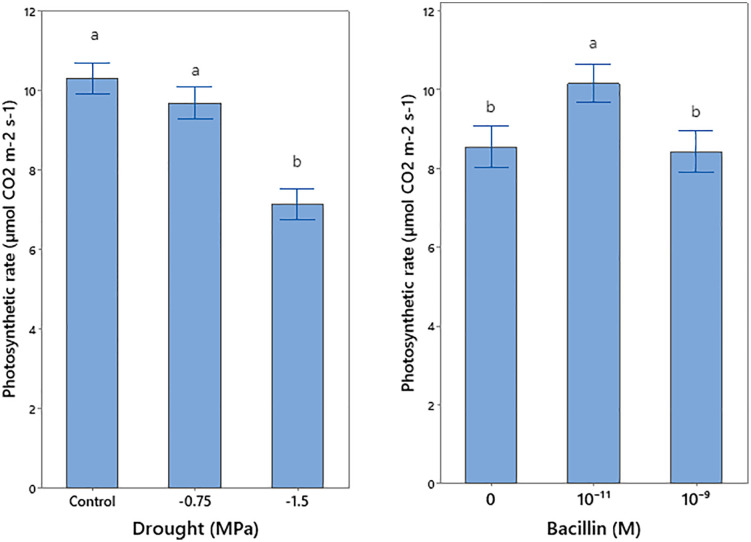
Effect of drought stress and Bacillin 20 on soybean photosynthetic rate. Means that do not share a letter are significantly different according to a Tukey test with **p* *≤ 0.05. Bars represent standard errors.

**Fig 5:**
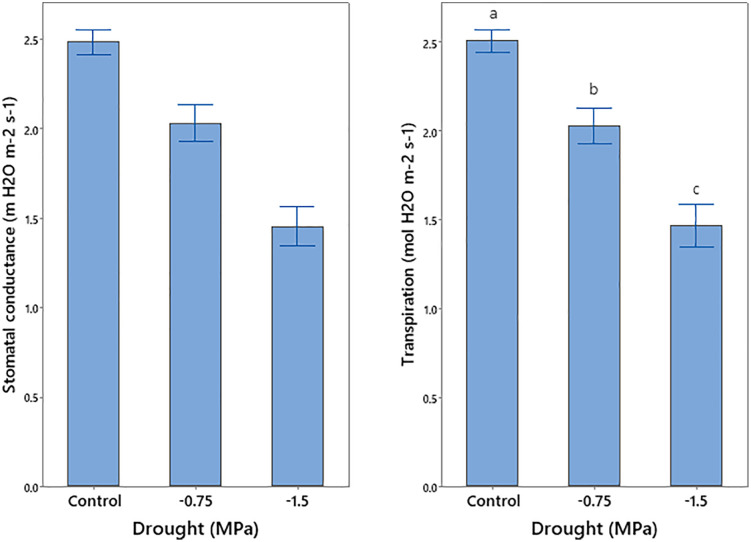
Effect of drought stress on soybean stomatal conductance and transpiration. Means that do not share a letter are significantly different according to a Tukey test with **p* *≤ 0.05. Bars represent standard errors.

**Fig 6:**
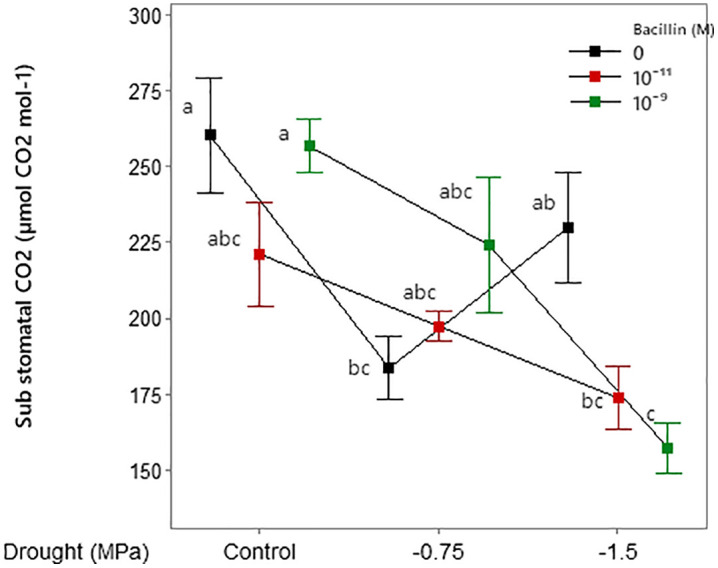
Effects of drought stress and Bacillin 20 on soybean sub stomatal CO_2_. Means that do not share a letter are significantly different according to a Tukey test with *p* ≤ 0.05. Bars represent standard errors.

Drought stress and Bacillin 20 application significantly affected photosynthetic rate (Table 1). Severe drought (−1.5 MPa) reduced photosynthetic rate, while Bacillin 20 at 10^−11^ M increased photosynthetic rate under both control and drought conditions (Fig 4b). Stomatal conductance and transpiration were significantly reduced by drought (*p* ≤ 0.01, Table 1), with greater decreases under severe drought (Fig 5). The interaction between drought and Bacillin 20 was significant for substomatal CO_2_ concentration (*p* ≤ 0.05, Table 1). Drought stress decreased substomatal CO_2_, while Bacillin 20 application showed varying trends. Under severe drought (−1.5 MPa), Bacillin 20 (10^−9^ M) reduced substomatal CO_2_, whereas under control conditions, no significant effect was observed (Fig 6).

### Leaf nutrient content

**Fig 7:**
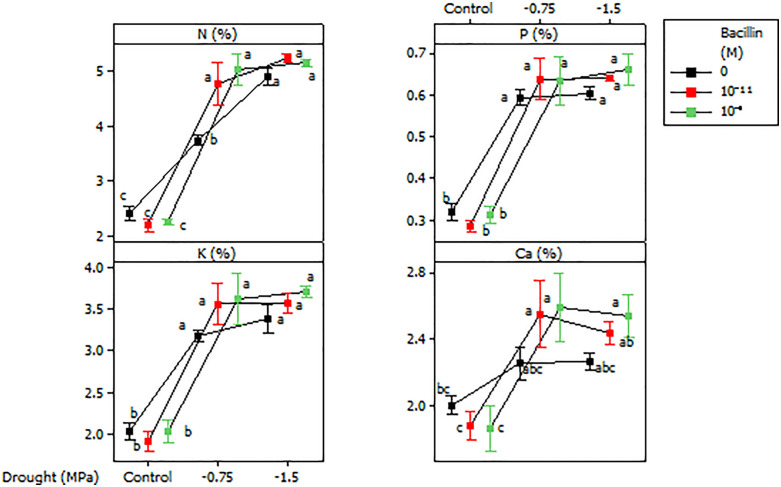
Effects of drought and Bacillin 20 on soybean leaf N, P, K, and Ca contents. Bars represent standard errors. Means that do not share a letter are significantly different according to a Tukey test with *p* ≤ 0.05. Bars represent standard errors.

**Fig 8:**
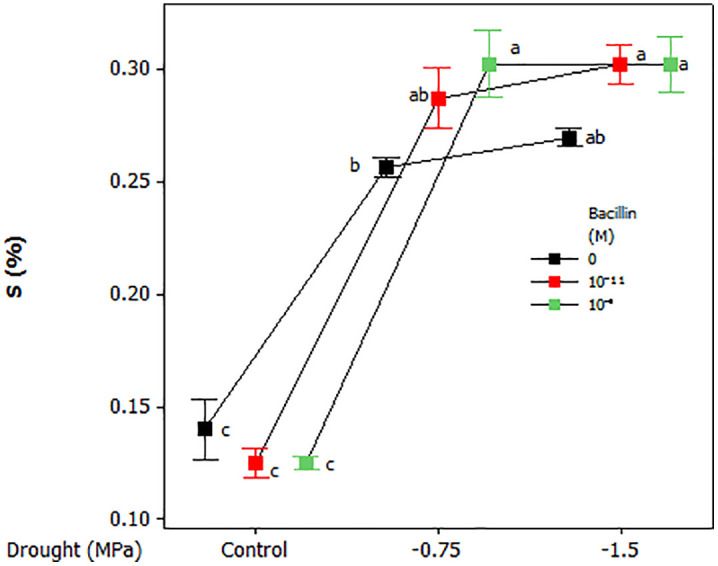
Effects of drought and Bacillin 20 on soybean leaf S content. Bars represent standard errors. Means that do not share a letter are significantly different according to a Tukey test with *p* ≤ 0.05. Bars represent standard errors.

Leaf nutrient content was influenced by drought and Bacillin 20. An interaction between these factors was observed for leaf N content, with lower N levels under control conditions compared to drought stress (Fig 7). Bacillin 20 application (both concentrations) increased leaf N at −0.75 MPa but had no effect under control or severe drought (−1.5 MPa) conditions. Leaf P, K, and Ca were affected only by drought, with higher concentrations under drought compared to control conditions, while Bacillin 20 had no effect (Fig 7). Leaf S content increased under drought, and Bacillin 20 (10^−11^ M) further enhanced S levels at −0.75 MPa, but not at −1.5 MPa (Fig 8).

**Fig 9:**
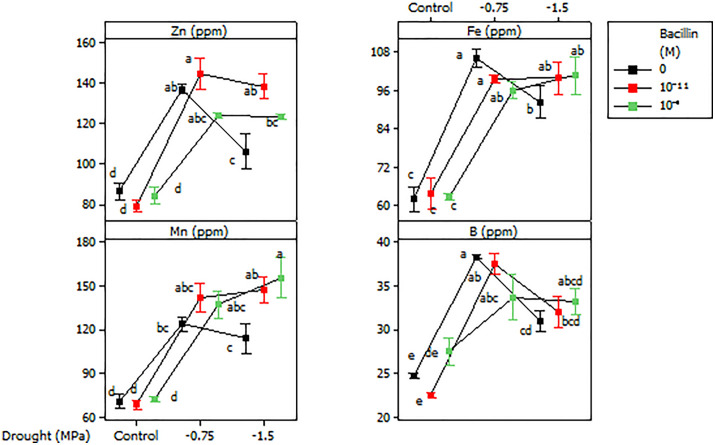
Effects of drought and Bacillin 20 on soybean leaf Zn, Fe, Mn and B contents. Bars represent standard errors. Means that do not share a letter are significantly different according to a Tukey test with *p* ≤ 0.05. Bars represent standard errors.

Leaf Mg, Zn, Fe, Mn, and B contents were also affected by drought and Bacillin 20. Drought increased leaf Mg content (Table 2), but Bacillin 20 had no effect. Similarly, Zn, Fe, Mn, and B increased under drought (Fig 9). Bacillin 20 effects varied across elements; at −1.5 MPa, 10^−9^ M Bacillin 20 reduced Zn and Mn levels, whereas no significant effect was observed at other drought levels (Fig 9).

## Discussion

The results demonstrate the profound effects of drought stress on soybean physiology, including reductions in plant height, leaf area, shoot biomass, photosynthetic parameters, and root architecture. These declines align with well-documented drought responses where plants prioritize water conservation over growth [25,26]. Under moderate drought, partial stomatal closure and osmotic adjustments enable sustained photosynthesis through improved mesophyll conductance and antioxidant activity [27,28]. However, severe drought overwhelms these compensatory mechanisms, leading to irreversible metabolic dysfunction.

Bacillin 20 mitigated drought effects by enhancing leaf area, shoot biomass, and root tip proliferation. As a bacterial signal compound, it likely activates stress-responsive pathways that overlap with microbial-associated molecular patterns (MAMPs) [5,29]. A growing body of evidence highlights the relevance of biostimulants in enhancing crop resilience to abiotic stresses such as drought. Di Sario et al. emphasized that plant biostimulants, including microbial derivatives, can regulate physiological and molecular responses in crops, improving stress tolerance, water-use efficiency, and overall performance under adverse conditions [30]. Biostimulants enhance plant resilience under drought and heat stress by modulating stress-responsive pathways, boosting antioxidant defenses, and promoting the accumulation of osmolytes, thereby supporting photosynthetic activity and water use efficiency under limited water availability [31].

It has been characterized for its bio-stimulatory effects on plant growth under optimal and stressful conditions in *Arabidopsis* [29], soybean [6,11,12], and corn [12]. The drought mitigation effects of Bacillin 20 may be due to increases in proline content and changes in levels of drought-specific ribosomal proteins, glutathione S-transferase, late embryogenesis proteins, vegetative storage proteins 1 and 2, thaumatin-like proteins, and proteins related to chloroplast and carbon metabolism [32]. Root Plasticity also may be affected by Bacillin 20. This compound promotes root elongation and branching via auxin-like signaling [11], expanding the rhizosphere volume for water/nutrient foraging in dry soils.

Contrary to studies reporting nutrient depletion under drought [3,33], this study observed increased leaf nutrient concentrations, likely due to passive solute concentration and active drought-induced uptake mechanisms. However, Bacillin 20 further modulated these dynamics in a nutrient-specific manner, suggesting targeted physiological interventions rather than generalized effects.

The drought-driven rise in leaf N aligns with heightened demand for stress-related proteins [34]. Thuricin 17 enhanced N assimilation under moderate drought (−0.75 MPa), potentially via upregulation of nitrate reductase or ammonium transporters [35]. Under severe stress (−1.5 MPa), however, root hydraulic failure likely limited Bacillin 20 efficacies, reflecting threshold-dependent biostimulant activity.

Elevated P, K, and Ca under drought reflect their roles in energy metabolism, osmotic regulation, and stress signaling [36,37]. The absence of Bacillin 20 effects on these macronutrients implies that drought-driven physiological adjustments (e.g., membrane remodeling, solute accumulation) dominate their homeostasis, overshadowing biostimulant-mediated pathways.

Drought-induced S accumulation supports glutathione synthesis for ROS detoxification [38]. Bacillin 20 amplified S uptake at 10 ⁻ ¹¹ M under moderate drought, possibly by stimulating sulfate transporter expression or enhancing ATP sulfurylase activity, synergizing with drought-triggered antioxidant demands [39].

Drought stress and Bacillin 20 application significantly altered leaf micronutrient profiles, with magnesium (Mg), zinc (Zn), iron (Fe), manganese (Mn), and boron (B) exhibiting distinct responses. Notably, drought increased Mg content in leaves, likely reflecting its indispensable role in sustaining photosynthetic machinery and ribosomal stability under water deficit [40]. However, Bacillin 20 had no discernible effect on Mg levels, suggesting that Mg homeostasis is stringently regulated and resistant to biostimulant-induced perturbations under drought. In contrast, Zn and Mn accumulation displayed dose-dependent interactions with Bacillin 20. While severe drought typically elevates micronutrient concentrations due to reduced transpiration-driven dilution [41], the application of Bacillin 20 at 10 ⁻ ⁹ M under severe drought paradoxically reduced Zn and Mn levels. This could arise from Bacillin 20-induced shifts in root exudate composition, such as altered organic acid secretion or rhizosphere pH gradients, which limit Zn/Mn bioavailability [42], or enhanced vacuolar sequestration in roots to mitigate metal toxicity under extreme stress. Conversely, at optimal doses, Bacillin 20 likely enhances Zn/Mn uptake by upregulating ZIP (Zn-regulated transporter) and NRAMP (Natural Resistance-Associated Macrophage Protein) transporters through nitric oxide (NO)-mediated signaling [43], and solubilizing soil-bound Zn/Mn oxides via H ⁺ -ATPase-driven rhizosphere acidification [44]. These findings highlight the context-specific interplay between Bacillin 20 dosage and drought severity in modulating micronutrient dynamics.

Overall, the observed changes in leaf nutrient concentrations suggest that drought stress significantly alters nutrient dynamics, while Bacillin 20 interacts with these processes in a nutrient-specific manner. Further studies are needed to explore the mechanisms by which Bacillin 20 influences micronutrient homeostasis under water-deficit conditions.

## Conclusions

In conclusion, the findings of this study contribute to our understanding of the intricate responses of soybean plants to drought stress and Bacillin 20 application. The results highlight the potential of *Bacillus*-based bio-stimulants as a sustainable approach to enhance plant resilience and productivity under water-limited environments. The study demonstrates that drought stress significantly affects leaf nutrient content, and Bacillin 20 application can modulate these effects, particularly under moderate drought conditions. The differential responses observed underscore the need for a nuanced understanding of how biostimulants interact with plant physiological processes under varying environmental stresses.

Given the increasing frequency and intensity of drought events due to climate change, the use of biostimulants such as Bacillin 20 offers a practical tool for building more climate-resilient cropping systems. Further studies should explore the underlying mechanisms to optimize the use of biostimulants such as Bacillin 20 for improving plant resilience to drought, particularly given that the incidence of drought conditions is likely to increase as climate change conditions further develop.

## Supporting information

S1 Data. (XLSX)

## References

[1] Wang S, Liu S, Wang J, Yokosho K, Zhou B, Yu Y-C, et al. Simultaneous changes in seed size, oil content and protein content driven by selection of SWEET homologues during soybean domestication. Natl Sci Rev. 2020;7(11):1776–86. doi: 10.1093/nsr/nwaa110 34691511 PMC8290959

[2] Joorabi S, Eisvand HR, Ismaili A, Nasrolahi AH. Effects of Zn nano-chelate foliar application on some physiological parameters and grain yield of soybean under water deficit stress. Plant Process and Function. 2020;9(35):73–86.

[3] Bista DR, Heckathorn SA, Jayawardena DM, Mishra S, Boldt JK. Effects of Drought on Nutrient Uptake and the Levels of Nutrient-Uptake Proteins in Roots of Drought-Sensitive and -Tolerant Grasses. Plants (Basel). 2018;7(2):28. doi: 10.3390/plants7020028 29601475 PMC6027393

[4] Zia R, Nawaz MS, Siddique MJ, Hakim S, Imran A. Plant survival under drought stress: Implications, adaptive responses, and integrated rhizosphere management strategy for stress mitigation. Microbiol Res. 2021;242:126626. doi: 10.1016/j.micres.2020.126626 33189069

[5] Nazari M, Smith DL. A PGPR-Produced Bacteriocin for Sustainable Agriculture: A Review of Thuricin 17 Characteristics and Applications. Front Plant Sci. 2020;11:916. doi: 10.3389/fpls.2020.00916 32733506 PMC7358586

[6] Subramanian S, Ricci E, Souleimanov A, Smith DL. A Proteomic Approach to Lipo-Chitooligosaccharide and Thuricin 17 Effects on Soybean GerminationUnstressed and Salt Stress. PLoS One. 2016;11(8):e0160660. doi: 10.1371/journal.pone.0160660 27560934 PMC4999219

[7] Alori ET, Onaolapo AO, Ibaba AL. Cell free supernatant for sustainable crop production. Front Sustain Food Syst. 2025;9. doi: 10.3389/fsufs.2025.1549048

[8] Rizza A, Tang B, Stanley CE, Grossmann G, Owen MR, Band LR, et al. Differential biosynthesis and cellular permeability explain longitudinal gibberellin gradients in growing roots. Proc Natl Acad Sci U S A. 2021;118(8):e1921960118. doi: 10.1073/pnas.1921960118 33602804 PMC7923382

[9] Fiodor A, Singh S, Pranaw K. The Contrivance of Plant Growth Promoting Microbes to Mitigate Climate Change Impact in Agriculture. Microorganisms. 2021;9(9):1841. doi: 10.3390/microorganisms9091841 34576736 PMC8472176

[10] Gray EJ, Lee KD, Souleimanov AM, Di Falco MR, Zhou X, Ly A, et al. A novel bacteriocin, thuricin 17, produced by plant growth promoting rhizobacteria strain Bacillus thuringiensis NEB17: isolation and classification. J Appl Microbiol. 2006;100(3):545–54. doi: 10.1111/j.1365-2672.2006.02822.x 16478494

[11] Bai Y, Zhou X, Smith DL. Enhanced Soybean Plant Growth Resulting from Coinoculation of Bacillus Strains with Bradyrhizobium japonicum. Crop Science. 2003;43(5):1774–81. doi: 10.2135/cropsci2003.1774

[12] Lee KD, Gray EJ, Mabood F, Jung W-J, Charles T, Clark SRD, et al. The class IId bacteriocin thuricin-17 increases plant growth. Planta. 2009;229(4):747–55. doi: 10.1007/s00425-008-0870-6 19083012

[13] Eisvand HR, Kamaei H, Nazarian F. Chlorophyll fluorescence, yield and yield components of bread wheat affected by phosphate bio-fertilizer, zinc and boron under late-season heat stress. Photosynt. 2018;56(4):1287–96. doi: 10.1007/s11099-018-0829-1

[14] Monjezi N, Yaghoubian I, Smith DL. Cell-free supernatant of Devosia sp. (strain SL43) mitigates the adverse effects of salt stress on soybean (Glycine max L.) seed vigor index. Front Plant Sci. 2023;14:1071346. doi: 10.3389/fpls.2023.1071346 37056501 PMC10086148

[15] Eisvand HR, Dousti A, Majnoun Hosseini N, Pour Babaie A. Effects of PGPR bacteria and seed ageing on improving common bean (Phaseolus vulgaris L.) yield and yield components. Iranian Journal of Field Crop Science. 2014;45(2):277–85. doi: 10.22059/ijfcs.2014.51906

[16] Naamala J, Msimbira LA, Subramanian S, Smith DL. Lactobacillus helveticus EL2006H cell-free supernatant enhances growth variables in Zea mays (maize), Glycine max L. Merill (soybean) and Solanum tuberosum (potato) exposed to NaCl stress. Front Microbiol. 2023;13:1075633. doi: 10.3389/fmicb.2022.1075633 36704564 PMC9871818

[17] Kang S-M, Radhakrishnan R, Khan AL, Kim M-J, Park J-M, Kim B-R, et al. Gibberellin secreting rhizobacterium, Pseudomonas putida H-2-3 modulates the hormonal and stress physiology of soybean to improve the plant growth under saline and drought conditions. Plant Physiol Biochem. 2014;84:115–24. doi: 10.1016/j.plaphy.2014.09.001 25270162

[18] Prudent M, Salon C, Souleimanov A, Emery RJN, Smith DL. Soybean is less impacted by water stress using Bradyrhizobium japonicum and thuricin-17 from Bacillus thuringiensis. Agron Sustain Dev. 2014;35(2):749–57. doi: 10.1007/s13593-014-0256-z

[19] Latifinia E, Eisvand HR. Soybean Physiological Properties and Grain Quality Responses to Nutrients, and Predicting Nutrient Deficiency Using Chlorophyll Fluorescence. J Soil Sci Plant Nutr. 2022;22(2):1942–54. doi: 10.1007/s42729-022-00785-0

[20] Boyer JS. Plant productivity and environment. Science. 1982;218(4571):443–8. doi: 10.1126/science.218.4571.443 17808529

[21] Manavalan LP, Guttikonda SK, Tran L-S, Nguyen HT. Physiological and molecular approaches to improve drought resistance in soybean. Plant Cell Physiol. 2009;50(7):1260–76. doi: 10.1093/pcp/pcp082 19546148

[22] Nazari M, Smith DL. Mitigation of drought or a combination of heat and drought stress effects on canola by Thuricin 17, a PGPR-produced compound. Frontiers in Sustainable Food Systems. 2023;7:1237206.

[23] Subramanian S, Souleimanov A, Smith DL. Thuricin17 Production and Proteome Differences in Bacillus thuringiensis NEB17 Cell-Free Supernatant Under NaCl Stress. Front Sustain Food Syst. 2021;5. doi: 10.3389/fsufs.2021.630628

[24] Michel BE. Evaluation of the Water Potentials of Solutions of Polyethylene Glycol 8000 Both in the Absence and Presence of Other Solutes. Plant Physiol. 1983;72(1):66–70. doi: 10.1104/pp.72.1.66 16662983 PMC1066170

[25] Bashir SS, Hussain A, Hussain SJ, Wani OA, Zahid Nabi S, Dar NA, et al. Plant drought stress tolerance: understanding its physiological, biochemical and molecular mechanisms. Biotechnology & Biotechnological Equipment. 2021;35(1):1912–25. doi: 10.1080/13102818.2021.2020161

[26] Gebre MG, Earl HJ. Effects of Growth Medium and Water Stress on Soybean [Glycine max (L.) Merr.] Growth, Soil Water Extraction and Rooting Profiles by Depth in 1-m Rooting Columns. Front Plant Sci. 2020;11:487. doi: 10.3389/fpls.2020.00487 32508851 PMC7250135

[27] Flexas J, Bota J, Cifre J, Mariano Escalona J, Galmés J, Gulías J, et al. Understanding down‐regulation of photosynthesis under water stress: future prospects and searching for physiological tools for irrigation management. Annals of Applied Biology. 2004;144(3):273–83. doi: 10.1111/j.1744-7348.2004.tb00343.x

[28] Chaves MM, Maroco JP, Pereira JS. Understanding plant responses to drought - from genes to the whole plant. Funct Plant Biol. 2003;30(3):239–64. doi: 10.1071/FP02076 32689007

[29] Subramanian S, Souleimanov A, Smith DL. Proteomic Studies on the Effects of Lipo-Chitooligosaccharide and Thuricin 17 under Unstressed and Salt Stressed Conditions in Arabidopsis thaliana. Front Plant Sci. 2016;7:1314. doi: 10.3389/fpls.2016.01314 27625672 PMC5003918

[30] Di Sario L, Boeri P, Matus JT, Pizzio GA. Plant Biostimulants to Enhance Abiotic Stress Resilience in Crops. Int J Mol Sci. 2025;26(3):1129. doi: 10.3390/ijms26031129 39940896 PMC11817731

[31] Carillo P. Can biostimulants enhance plant resilience to heat and water stress in the Mediterranean hotspot?. Plant Stress. 2025;16:100802. doi: 10.1016/j.stress.2025.100802

[32] Subramanian S, Mitkus E, Souleimanov A, Smith DL. Lipo-chitooligosaccharide and thuricin 17 act as plant growth promoters and alleviate drought stress in Arabidopsis thaliana. Front Microbiol. 2023;14:1184158. doi: 10.3389/fmicb.2023.1184158 37601342 PMC10436337

[33] Yang X, Lu M, Wang Y, Wang Y, Liu Z, Chen S. Response Mechanism of Plants to Drought Stress. Horticulturae. 2021;7(3):50. doi: 10.3390/horticulturae7030050

[34] Nayyar H, Gupta D. Differential sensitivity of C3 and C4 plants to water deficit stress: Association with oxidative stress and antioxidants. Environmental and Experimental Botany. 2006;58(1–3):106–13. doi: 10.1016/j.envexpbot.2005.06.021

[35] Calvo P, Nelson L, Kloepper JW. Agricultural uses of plant biostimulants. Plant Soil. 2014;383(1–2):3–41. doi: 10.1007/s11104-014-2131-8

[36] Farooq M, Wahid A, Kobayashi N, Fujita D, Basra SMA. Plant drought stress: effects, mechanisms and management. Agron Sustain Dev. 2009;29(1):185–212. doi: 10.1051/agro:2008021

[37] Wang M, Zheng Q, Shen Q, Guo S. The critical role of potassium in plant stress response. Int J Mol Sci. 2013;14(4):7370–90. doi: 10.3390/ijms14047370 23549270 PMC3645691

[38] Hasanuzzaman M, Nahar K, Anee TI, Fujita M. Glutathione in plants: biosynthesis and physiological role in environmental stress tolerance. Physiol Mol Biol Plants. 2017;23(2):249–68. doi: 10.1007/s12298-017-0422-2 28461715 PMC5391355

[39] Anjum NA, Gill R, Kaushik M, Hasanuzzaman M, Pereira E, Ahmad I, et al. ATP-sulfurylase, sulfur-compounds, and plant stress tolerance. Front Plant Sci. 2015;6:210. doi: 10.3389/fpls.2015.00210 25904923 PMC4387935

[40] Cakmak I, Yazici AM. Magnesium: a forgotten element in crop production. Better crops with plant food. 2010;94:23–5.

[41] Thapa S, Bhandari A, Ghimire R, Xue Q, Kidwaro F, Ghatrehsamani S, et al. Managing Micronutrients for Improving Soil Fertility, Health, and Soybean Yield. Sustainability. 2021;13(21):11766. doi: 10.3390/su132111766

[42] Halpern M, Bar-Tal A, Ofek M, Minz D, Muller T, Yermiyahu U. The use of biostimulants for enhancing nutrient uptake. In: Sparks DL, editor. Advances in Agronomy. Academic Press; 2015. p. 141–74.

[43] Buet A, Galatro A, Ramos-Artuso F, Simontacchi M. Nitric oxide and plant mineral nutrition: current knowledge. J Exp Bot. 2019;70(17):4461–76. doi: 10.1093/jxb/erz129 30903155

[44] Santi S, Schmidt W. Dissecting iron deficiency‐induced proton extrusion in Arabidopsis roots. New Phytologist. 2009;183(4):1072–84. doi: 10.1111/j.1469-8137.2009.02908.x 19549134

